# Designing of Co_0.5_Ni_0.5_Ga_x_Fe_2−x_O_4_ (0.0 ≤ x ≤ 1.0) Microspheres via Hydrothermal Approach and Their Selective Inhibition on the Growth of Cancerous and Fungal Cells

**DOI:** 10.3390/pharmaceutics13070962

**Published:** 2021-06-26

**Authors:** Suriya Rehman, Munirah A. Almessiere, Suhailah S. Al-Jameel, Uzma Ali, Yassine Slimani, Nedaa Tashkandi, Najat S. Al-Saleh, Ayyar Manikandan, Firdos Alam Khan, Ebtesam A. Al-Suhaimi, Abdulhadi Baykal

**Affiliations:** 1 Department of Epidemic Diseases Research, Institute for Research & Medical Consultations (IRMC), Imam Abdulrahman Bin Faisal University, Dammam 31441, Saudi Arabia; 2 Department of Biophysics, Institute for Research and Medical Consultations, Imam Abdulrahman Bin Faisal University, P.O. Box 1982, Dammam 31441, Saudi Arabia; malmessiere@iau.edu.sa (M.A.A.); yaslimani@iau.edu.sa (Y.S.); 3 Department of Physics, College of Science, Imam Abdulrahman Bin Faisal University, Dammam 31441, Saudi Arabia; 4 Department of Chemistry, College of Science, Imam Abdulrahman Bin Faisal University, Dammam 31441, Saudi Arabia; ssaljameel@iau.edu.sa; 5 Department of Public Health, College of Public Health, Imam Abdulrahman Bin Faisal University, Dammam 31441, Saudi Arabia; uasali@iau.edu.sa; 6 Department of Nanomedicine, Institute for Research and Medical Consultations, Imam Abdulrahman Bin Faisal University, P.O. Box 1982, Dammam 31441, Saudi Arabia; natashkandi@iau.edu.sa (N.T.); abaykal@iau.edu.sa (A.B.); 7 Family and Community Medicine, King Fahad Hospital of the University, Imam Abdulrahman Bin Faisal University, P.O. Box 1982, Dammam 31441, Saudi Arabia; nssaleh@iau.edu.sa; 8 Department of Chemistry, Bharath Institute of Higher Education and Research (BIHER), Bharath University, Chennai 600 073, Tamil Nadu, India; maikandan@gmail.com; 9 Department of Stem Cell Research, Institute for Research and Medical Consultations (IRMC), Imam Abdulrahman Bin Faisal University, P.O. Box 1982, Dammam 31441, Saudi Arabia; fakhan@iau.edu.sa; 10 Biology Department, College of Science, Imam Abdulrahman Bin Faisal University, P.O. Box 1982, Dammam 31441, Saudi Arabia; ealsuhaimi@iau.edu.sa

**Keywords:** nanomaterial synthesis, anti-cancer agents, anti-fungal, microspheres

## Abstract

The current study offers an efficient design of novel nanoparticle microspheres (MCs) using a hydrothermal approach. The Co_0.5_Ni_0.5_Ga_x_Fe_2−x_O_4_ (0.0 ≤ x ≤ 1.0) MCs were prepared by engineering the elements, such as cobalt (Co), nickel (Ni), iron (Fe), and gallium (Ga). There was a significant variation in MCs’ physical structure and surface morphology, which was evaluated using energy dispersive X-ray analysis (EDX), X-ray diffractometer (XRD), high-resolution transmission electron microscopy (HR-TEM), and scanning electron microscope (SEM). The anti-proliferative activity of MCs was examined by MTT assay and DAPI staining using human colorectal carcinoma cells (HCT-116), human cervical cancer cells (HeLa), and a non-cancerous cell line—human embryonic kidney cells (HEK-293). Post 72 h treatment, MCs caused a dose dependent inhibition of growth and proliferation of HCT-116 and HeLa cells. Conversely, no cytotoxic effect was observed on HEK-293 cells. The anti-fungal action was assessed by the colony forming units (CFU) technique and SEM, resulting in the survival rate of *Candida albicans* as 20%, with severe morphogenesis, on treatment with MCs x = 1.0. These findings suggest that newly engineered microspheres have the potential for pharmaceutical importance, in terms of infectious diseases and anti-cancer therapy.

## 1. Introduction

Nanomaterials are promising materials for various therapeutic applications, especially in diagnosing and treating diverse types of cancers and infectious diseases. Metal-based treatments are known to be an attractive research field in medicinal chemistry. Although platinum-based therapy is currently used, it is accompanied by resistance, deterrent side effects and a deficit of selectivity, forcing researchers to find more safe metals [1]. For example, complexes of nano-inorganic metals, such as Ru, Ir, Cu, Ni, Zn, Co, etc., were found to have better anti-cancer properties than cisplatin [2,3]. These nanomaterials possess unique properties, like high surface area and better cell penetration capability [4]. Among them, spinel ferrite nanomaterials are the most preferred materials for biomedicine, magnetic resonance imaging, pharmaceuticals, sensors, and drug delivery [5,6,7,8]. The ecofriendly transition metal oxides, such as nickel (Ni), cobalt (Co), and manganese (Mn), are known to exhibit superior redox activity, with natural richness, and simplistic and scalable synthesis [9,10,11,12]. There are reports that suggest that treatment with nickel nanoparticles can induce anti-cancer activities in different types of cancer cells [13,14,15,16]. Cobalt nanoparticles also possess anti-cancer activities, as shown in several studies [17,18,19,20]. Besides, gallium nanoparticles also show anti-cancer drug delivery and cancer cell imaging capabilities [21,22,23]. Iron oxide nanoparticles are also applied for cancer cell imaging and treatments [24,25,26].

Previously, it has been shown that combining two or more nanoparticles is an effective strategy to synthesize nanocomposites for targeted drug delivery and anti-cancer treatment [27,28,29,30]. These data support the evidence that combining two or more nanoparticles enhances the anti-cancer activities in colon and breast cancer cells.

The microsphere structure has attracted more attention due to low density, good dispersion, high specific surface area, high surface activity, strong permeability, good stability, super paramagnetic, etc. [31,32,33,34]. Some researchers reported that the CoFe_2_O_4_ microspheres have the capability to attach to negatively charged bacterial pathogens (e.g., *E. coli*) [35]. Others found that the CoFe2O4 microspheres in many samples actively target *E. coli*, and immobilize it on their surface with an efficiency of 90% [36] (Huang et al. 2016). Li et al. investigated the anti-bacterial effectiveness and found that CoFe2O4 nanoparticles can act on gram-negative bacteria at lower concentrations within 1 h [37]. Taking into consideration the advantage of CoFe2O4 microspheres for its ability to attach to negatively charged bacterial cells, Muruganantham et al. fabricated positively charged CoFe2O4 microspheres as biocompatible anode in microbial cells for power generation [38]. Likewise, Ping Chen et al. produced M_x_Fe_3−x_O_4_ (M = Mg, Mn, Fe, Co, Ni, Cu, Zn) microspheres for magnetic targeting and microwave heating, that can therefore be used for targeted and controllable drug delivery [39].

In the present study, the elements such as cobalt, nickel, gallium, and iron oxide were combined, and gallium was substituted with cobalt and nickel, to prepare Co_0.5_Ni_0.5_Ga_x_Fe_2−x_O_4_ (0.0 ≤ x ≤ 1.0) MCs by hydrothermal approach. The MCs structure and morphology were evaluated by using XRD, EDX, TEM, and SEM techniques. The impact of Co_0.5_Ni_0.5_Ga_x_Fe_2−x_O_4_ (0.0 ≤ x ≤ 1.0) MCs was examined using colon cancer HCT-116 and cervical cancer HeLa by using MTT assay. In addition, the anti-fungal effects of Co_0.5_Ni_0.5_Ga_x_Fe_2−x_O_4_ (0.0 ≤ x ≤ 1.0) MCs were also evaluated on *Candida* cells.

## 2. Materials and Methods

### 2.1. Synthesis and Characterizations of MCs

Carbon microspheres were prepared hydrothermally using 1M glucose aqueous solution in a Teflon-lined autoclave, at 180 °C for 10 h. The black solid was obtained by centrifuging, washed many times with deionized H_2_O and C_2_H_5_OH (ethanol), respectively, and then dried at 60 °C. For the synthesis of Co_0.5_Ni_0.5_Ga_x_Fe_2−x_O_4_ (0.0 ≤ x ≤ 1.0) MCs, the stoichiometric amount of carbon sphere template and nitrate salts of Fe, Co, Ni, and Ga were dissolved in 50 mL of deionized water under continuous stirring, followed by sonication for approximately 30 min. Afterwards, the homogeneous solution was transferred into a Teflon-lined stainless steel autoclave and sealed to heat at 180 °C for 10 h. The final products were washed with distilled water and ethanol and dried at 60 °C. Finally, the solid product was heated at 500 °C for 4 h to get the Co_0.5_Ni_0.5_Ga_x_Fe_2−x_O_4_ (0.0 ≤ x ≤ 1.0) MCs. The characterization of synthesized nanomaterials was done as per the protocol by Almessiere et al. [30].

### 2.2. Anti-Cancer Activity

#### 2.2.1. Cell Culture and Testing of Nanoparticles Using MTT Method

Human colorectal carcinoma (HCT-116) and human cervical cells (HeLa) were taken for the study of the impact of Co_0.5_Ni_0.5_Ga_x_Fe_2−x_O_4_ (0.0 ≤ x ≤ 1.0) MCs on cell viability and cell proliferation. A non-cancer cell line, such as embryonic kidney cells (HEK-293), was used as control cell line and to examine the specificity of the MCs. As per the previously described method [40,41], the cells were cultured to 75–80% confluency and further processed using 3-(4,5-dimethylthiazol-2-yl)-2,5-diphenyl tetrazolium bromide (MTT) assay (Invitrogen, Waltham, MA, USA). The MTT assay was done where HCT-116, HeLa, and HEK-293 cells were treated with MCs doses, ranging from 2.0 to 80 µg/mL for 72 h. In the control group, no MCs were added. Both the control and sample-treated cells were exposed to 10 µL of MTT (5 mg/mL) and were incubated in a CO_2_ incubator for 4 h. Later, the cell culture media was replaced with DMSO (1%), and the plate was examined at a wavelength of 570 nm by using a Plate reader (BioTek Instruments, Winooski, VT, USA). The percentage of cell viability was calculated for the statistical analysis [42,43].

#### 2.2.2. DAPI Staining for DNA Analysis

DAPI (4′,6-diamidino-2-phenylindole) staining assay was done to examine cancer cell DNA. The cells were divided into two groups: the control group, in which no MCs were added, and the experimental group where Co_0.5_Ni_0.5_Ga_x_Fe_2−x_O_4_ (0.0 ≤ x ≤ 1.0) MCs (50 µg/mL) were added. Seventy-two hours post-treatment, both groups were exposed to paraformaldehyde and then washed with phosphate-buffered saline (PBS). Cells were then stained with DAPI for 5 min, and examined by using confocal scanning microscope (Zeiss, Jena, Germany) [42,43]. The data are presented as mean standard deviation obtained from triplicates and one-way ANOVA followed by Dunnett’s post hoc test with GraphPad Prism Software for final statistical analysis.

### 2.3. Anti-Candidal Activity

#### 2.3.1. Preparation of Inoculum and Nanomaterial

*Candida albicans* ATCC14053 (yeast) was chosen for the anti-fungal assay of the prepared Co_0.5_Ni_0.5_Ga_x_Fe_2−x_O_4_ (0.0 ≤ x ≤ 1.0) MCs. *C. albicans* was aerobically grown using Sabouraud broth (SDB), at 28 ± 2 °C for 24 h with shaking. Subsequently, the cell density was adjusted to approximately 10^7^ CFU/mL. For the nanomaterial sample preparation, a concentration of 20 mg/mL of synthesized MCs was used after sonication for 10 to 15 min, to get a suspended solution.

#### 2.3.2. Study of CFU and Morphogenesis of *C. albicans*

To demonstrate the anti-candida activity of MCs, colony-forming units (CFU) were used to evaluate the viable microbial cell concentrations in a sample, post treatment. Briefly, sterile tubes containing different concentrations of nanomaterial in SDB, were sonicated for 10 to 15 min to get the suspended solution. An adjusted inoculum of *C. albicans* was added to the MCs solution and incubated in a rotary shaker for 48 h at 28 ± 2 °C. Untreated *C. albicans* was used as a control. On the completion of the incubation period, 100 µL of culture was plated out onto the SDA plates and incubated as mentioned above. On the other day, the plates were observed and recorded for the number of colonies on each plate. Each colony counted is taken to have emerged from a single viable *Candida* cell. The plates having colonies above 400 were considered as a lawn culture. The survival percentage of *C. albicans* was calculated by the formula S% = (A/B) × 100 (where, A is the number of CFU in the medium treated with nanomaterial, and B is the number of CFU in the control) [44]. The fungal morphogenesis caused by the treatment of MCs was also studied by using SEM, following the protocol proposed by Aldakheel et al. [45]. The data are presented as mean standard deviation obtained from triplicates and one-way ANOVA followed by Dunnett’s post hoc test with GraphPad Prism Software, USA for final statistical analysis.

## 3. Results and Discussion

### 3.1. Microstructural Analysis of Co_0.5_Ni_0.5_Ga_x_Fe_2−x_O_4_ (0.0 ≤ x ≤ 1.0) MCs

**Figure 1:**
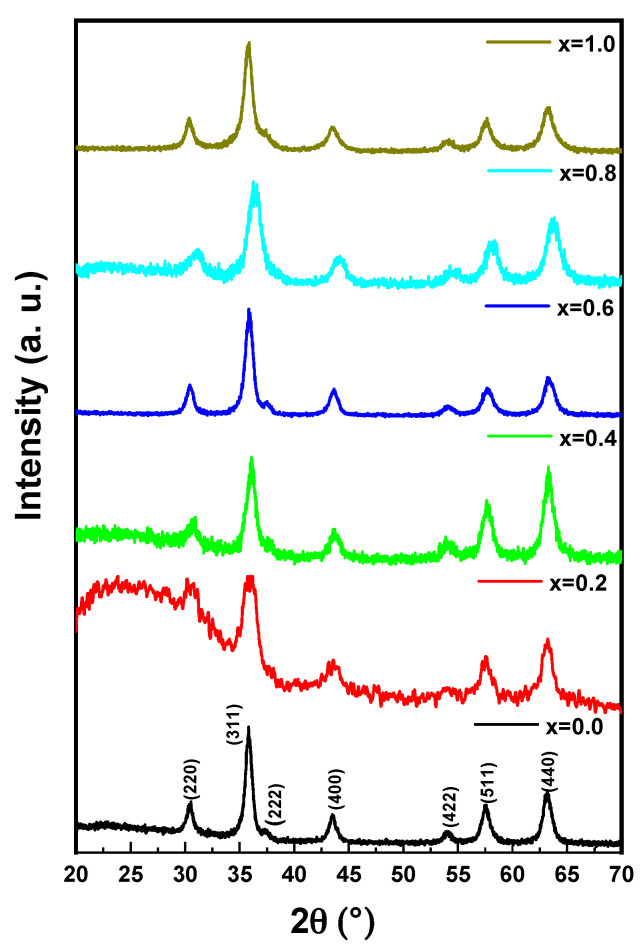
XRD powder patterns of Co_0.5_Ni_0.5_Ga_x_Fe_2−x_O_4_ (0.0 ≤ x ≤ 1.0) MCs.

**Figure 2:**
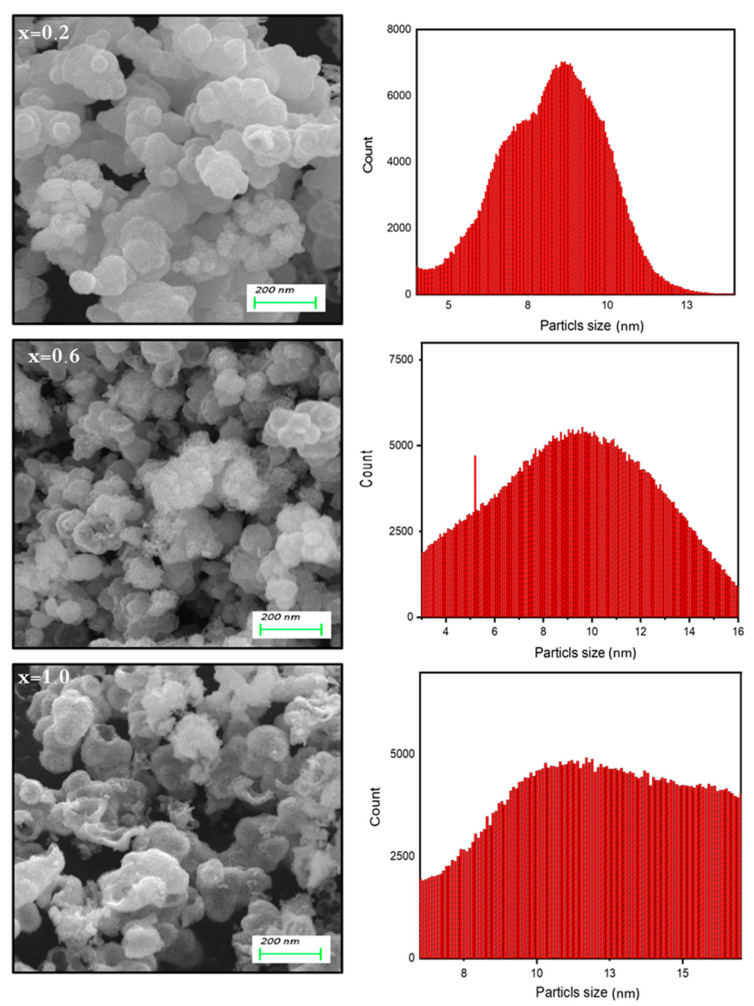
FESEM and particles size distribution of Co_0.5_Ni_0.5_Ga_x_Fe_2−x_O_4_ (x = 0.2, 0.6 and 1.0) MCs.

**Figure 3:**
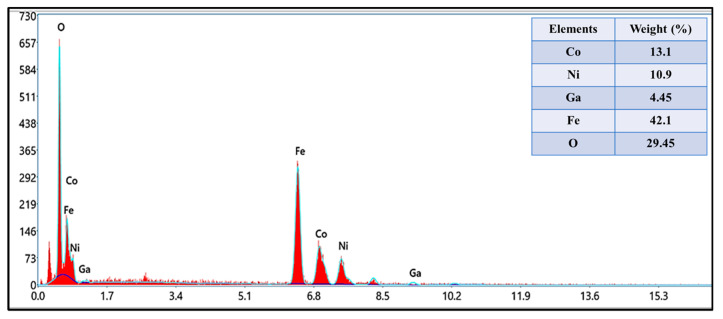
EDX of Co_0.5_Ni_0.5_Ga_x_Fe_2−x_O_4_ (x = 0.2) MCs.

**Figure 4:**
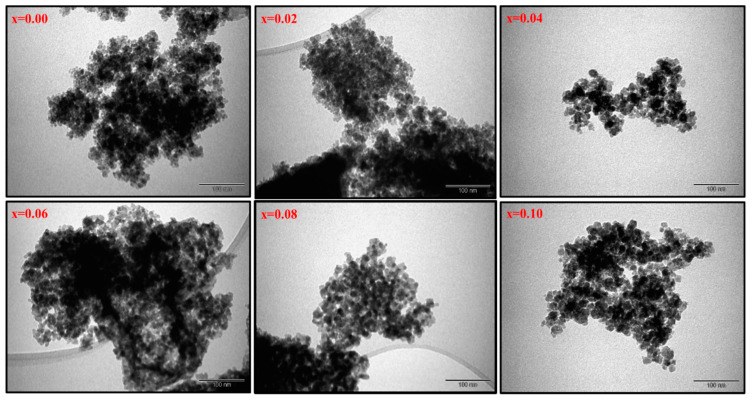
TEM images of Co_0.5_Ni_0.5_Ga_x_Fe_2−x_O_4_ (0.0 ≤ x ≤ 1.0) MCs.

The structure analysis of Co_0.5_Ni_0.5_Ga_x_Fe_2−x_O_4_ (0.0 ≤ x ≤ 1.0) MCs was offered through X-ray diffractometer, as seen in Figure 1. All MCs pattern denoted the index peaks of cubic spinel phase. Besides, the XRD patterns disclosed a pure spinel structure and slightly unsolicited phase. The cell parameters a, b, and c, were evaluated by full-proof software. It was found that the raise was with the growing amount of Ga from 5.883 (8) to 8.353 (5) (Å). The average crystallite size was measured by Scherrer’s equation and found to be around 20 ± 6 nm. Figure 2 exhibits the FESEM images of Co_0.5_Ni_0.5_Ga_x_Fe_2−x_O_4_ (x = 0.2, 0.6 and 1.0) MCs. The samples showed an accumulation of the cubic shape particles. The elemental composition (EDX) of Co_0.5_Ni_0.5_Ga_x_Fe_2−x_O_4_ (x = 0.2) MCs is shown in Figure 3. It approved the formation of MCs without any impurity. PDI was measured via zeta potential and the values were around 0.344, which indicated polydispersed particle size distribution. The TEM images demonstrated the agglomerated state of nanoparticles of MCs as seen in Figure 4.

### 3.2. Biological Activities

#### 3.2.1. Impact of Co_0.5_Ni_0.5_Ga_x_Fe_2−x_O_4_ (0.0 ≤ x ≤ 1.0) MCs on Cancer Cell Viability

The anti-proliferative impact of Co_0.5_Ni_0.5_Ga_x_Fe_2−x_O_4_ (0.0 ≤ x ≤ 1.0) MCs on both colon cancer (HCT-116) and cervical cancer (HeLa) cells was examined. The cell viability assay confirmed a significant decrease in the cell viability after the treatments of Co_0.5_Ni_0.5_Ga_x_Fe_2−x_O_4_ (0.0 ≤ x ≤ 1.0) MCs. The treatments of Co_0.5_Ni_0.5_Ga_x_Fe_2−x_O_4_ MCs (0.0 ≤ x ≤ 1.0) showed inhibitory action on cancer cell growth and proliferation. The calculated inhibitory concentration (IC_50_) of MCs was 25 µg/mL to 59 µg/mL for HCT-116 and 35 µg/mL to 60 µg/mL for HeLa cells. The impact of MCs on non-cancerous cells (HEK-293) was also tested. There was a decrease in the cancer viability, but the percentage of the decrease was not statistically significant (Table 1). Based on these observations, it may be suggested that the synthesized Co_0.5_Ni_0.5_Ga_x_Fe_2−x_O_4_ (0.0 ≤ x ≤ 1.0) MCs possess an inhibitory effect on HCT-116 and HeLa cells compared to HEK-293 cells. This is the first study that demonstrates the inhibitory effect on cell viability of synthesized Co_0.5_Ni_0.5_Ga_x_Fe_2−x_O_4_ (0.0 ≤ x ≤ 1.0) MCs against HCT-116 and HeLa cells.

We previously reported the impact of different nanomaterials on colon and breast cancer cells [45,46,47]. The presence of cobalt complexes in the nanocomposites may change the count and length of aliphatic chains, within the coordinated ligands. This significantly promotes its interaction with biological molecules, as well as its anti-proliferative effect on cancer cells [48]. Also, the apoptosis seen in the cancer cells may be attributed to the mode of action by Co II and Ni II, which was found with high anti-tumor effect by stimulating mitochondria-intermediated apoptosis, along with arresting the S-phase of the cell cycle [3].

#### 3.2.2. Disintegration of Cancer Cell DNA

**Figure 5:**
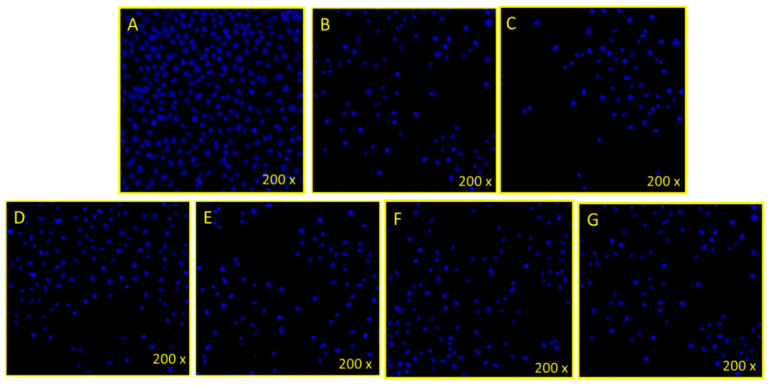
Cancer cell death via treatment of Co_0.5_Ni_0.5_Ga_x_Fe_2−x_O_4_ MCs: shows the impact of Co_0.5_Ni_0.5_Ga_x_Fe_2−x_O_4_ (0.0 ≤ x ≤ 1.0) MCs on cervical (HeLa) cancer cells stained with DAPI post 72 h treatment. Figure (**A**): control (without MCs treatment), (**B**): (0.0), (**C**): (0.02), (**D**): (0.04), (**E**): (0.06), (**F**): (0.08), and (**G**): (0.01).

The treatment of Co_0.5_Ni_0.5_Ga_x_Fe_2−x_O_4_ (0.0 ≤ x ≤ 1.0) MCs (50 µg/mL) caused a significant decrease in the number (35,000 to 45,000 cells/well) (Figure 5B–G) of HeLa cancer cells, compared to control cells (200,000 to 210,000 cells/well) (Figure 5A). The decrease in cancer cells was due to the mechanism of programmed cell death (known as apoptosis). The uptake of nanospheres by the treated cells also depends on the nanospheres concentrations, time of incubation, and other stress factors present [49]. The obtained results showed that the engineered microspheres have a cytotoxic effect on cancerous cells, through its magnetic effect, as MCs are supposed to localize into the target cell with the support of magnetic microspheres. These results agree with other studies where drug-loaded targeted magnetic microspheres are used for cancer therapy [50]. The mechanism of action of nanocomposites on cancer cells may due to ionic strength, magnetism, and other effects [51]. These factors may lead to changes in the permeability of cell membrane, dissolving the lipids, and fusion with the cell lysosomes. Such factors may cause cell death, leading to cell cytotoxicity.

#### 3.2.3. Anti-Fungal Activity

**Figure 6:**
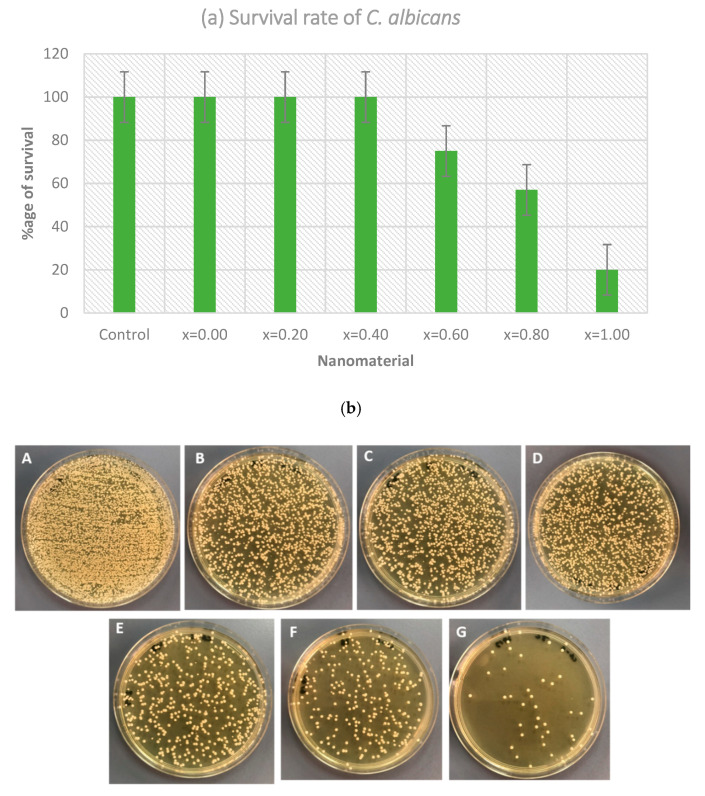
(**a**) Survival rate of *C. albicans* after the treatment with Co_0.5_Ni_0.5_Ga_x_Fe_2−x_O_4_ (0.0 ≤ x ≤ 1.0) MCs (*p* < 0.001); (**b**) Agar plates showing the CFU count of *C. albicans* after the treatment with Co_0.5_Ni_0.5_Ga_x_Fe_2−x_O_4_ MCs: (**A**) untreated; (**B**) x = 0.0; (**C**) x = 0.20; (**D**) x = 0.40; (**E**) x = 0.60; (**F**) x = 0.80; (**G**) x = 1.0.

The anti-candida action of MCs was assessed by CFU technique. The varying concentration of test material was taken into consideration. The survival percentage of *Candida* in the inoculated media added with nanomaterial was investigated as the potential of the synthesized microspheres. After plating the harvested treated culture on SDA plates for 24 h, the existing colonies of *Candida* were observed. The survival rate of *Candida* is shown in Figure 6a. It was observed that the survival rate of *Candida* was reduced with the increasing ratio of microspheres, i.e., the maximum effect was shown by x = 1.0 with a survival rate of 20%, followed by 0.8 and 0.6, with 57 and 75%, respectively. Other ratios were not found to be significantly useful and showed an approximately 100% survival rate, that was similar to control, i.e., the untreated *Candida*. As shown in Figure 6b, the number of cells on the agar plates has significantly decreased when compared to the control plate (untreated *Candida)*. This indicates that the increased ratios of microspheres had an impact on the growth of the organism, where maximum growth reduction was seen with samples x = 1.0, x = 0.8, and x = 0.6. Using one-way ANOVA, the variation in the survival rates of *C. albicans* when using a different concentration of nanomaterial was found to be significant (*p* < 0.001).

**Figure 7:**
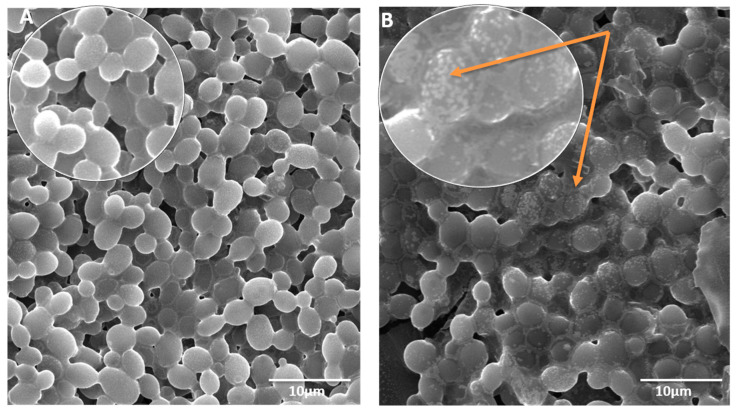
SEM micrograph presenting the morphogenesis of *C. albicans.* (**A**) Untreated cells (control); (**B**) MCs Treated cells.

Morphogenesis of *C. albicans* caused by the treatment of MCs was also studied by using SEM. The image (Figure 7) presents the morphological disruption caused by the MCs treatment. Figure 7B depicts the attachment and interaction of MCs and cellular surfaces, leading to disruption of *Candida* cell surface. The image clearly shows the membrane with attached MCs, creating perforations, whereas in Figure 7A, the untreated cells appear normal, with a smooth cell surface. The direct physical association of MCs and cells have been viewed, and the MCs have been observed to anchor onto cell walls and directly incorporate onto them. An apparent enhancement of anti-fungal activity was achieved on the different organizations of the metal ions. It is evident from the obtained results that the synthesized nanomaterial activity was also enhanced with further metal coordination. This elevation in the anti-fungal activity can be justified based on structures, carrying additional carbon, and nitrogen bonds. Additionally, chelation may have reduced the polarity of the metal ion by sharing positive charge partially with the donor groups. Therefore, this phenomenon enhances the lipophilic nature of the metal atom, hence favoring greater penetration through the cell wall of the *Candida* and thus inhibiting them significantly. Moreover, the dipole moment, solubility, and conductivity are also affected by several metal ions, which could also be the additional factors responsible for increasing the nanomaterial’s liposolubility, hence elevating the biological activity [29,52,53,54].

## 4. Conclusions

The Co_0.5_Ni_0.5_Ga_x_Fe_2−x_O_4_ (0.0 ≤ x ≤ 1.0) microspheres (MCs) were prepared using the hydrothermal approach, and the structures were confirmed using XRD, EDX, HR-TEM, TEM, and SEM techniques. The anti-proliferative impact of Co_0.5_Ni_0.5_Ga_x_Fe_2−x_O_4_ (0.0 ≤ x ≤ 1.0) MCs was examined by using MTT assay that showed a dose-dependent inhibition of cancer cells (HCT-116, and HeLa). Conversely, no cytotoxic effect was observed on the normal cell line (HEK-293). The treatment of Co_0.5_Ni_0.5_Ga_x_Fe_2−x_O_4_ (0.0 ≤ x ≤ 1.0) MCs also caused nuclear DNA disintegration in the cancer cells, as revealed by DAPI staining. Besides, the anti-fungal action was assessed by the CFU technique, and the survival rate of *C. albicans* was found to be reduced with the increasing ratio of Co_0.5_Ni_0.5_Ga_x_Fe_2−x_O_4_ (0.0 ≤ x ≤ 1.0) MCs. These findings suggest that the synthesized Co_0.5_Ni_0.5_Ga_x_Fe_2−x_O_4_ (0.0 ≤ x ≤ 1.0) MCs possess potential anti-fungal and anti-cancer capabilities, to be considered for potential pharmaceutical applications.

## Figures and Tables

**Table 1:** Impact of Co_0.5_Ni_0.5_Ga_x_Fe_2−x_O_4_ (0.0 ≤ x ≤ 1.0) MCs on cancerous and normal cell lines.

x	IC_50_ (HCT-116) Cells (µg/mL)	IC_50_ (HeLa) Cells (µg/mL)	IC_50_ (HEK-293) Cells
0.0	25 ± 2	35 ± 4	No inhibition
0.2	46 ± 3	56 ± 4	No inhibition
0.4	42 ± 4	48 ± 5	No inhibition
0.6	29 ± 2	50 ± 5	No inhibition
0.8	50 ± 5	49 ± 4	No inhibition
1.0	59 ± 5	60 ± 5	No inhibition

Inhibitory concentration (IC).

## Data Availability

Not applicable.

## References

[1] Dilruba S., Kalayda G.V. (2016). Platinum-based drugs: Past, present and future. Cancer Chemother. Pharmacol..

[2] Zeng L., Chen Y., Liu J., Huang H., Guan R., Ji L., Chao H. (2016). Ruthenium (II) complexes with 2-phenylimidazo [4,5-f][1,10] phenanthroline derivatives that strongly combat cisplatin-resistant tumor cells. Sci. Rep..

[3] Qin J.-L., Shen W.-Y., Chen Z.-F., Zhao L.-F., Qin Q.-P., Yu Y.-C., Liang H. (2017). Oxoaporphine Metal Complexes (Co^II^, Ni^II^, Zn^II^) with High Antitumor Activity by Inducing Mitochondria-Mediated Apoptosis and S-phase Arrest in HepG2. Sci. Rep..

[4] Tombuloglu H., Khan F.A., Almessiere M.A., Aldakheel S., Baykal A. (2020). Synthesis of niobium substituted cobalt-nickel nano-ferrite (Co_0.5_Ni_0.5_Nb_x_Fe_2−x_O_4_ (x ≤ 0.1) by hydrothermal approach show strong anti-colon cancer activities. J. Biomol. Struct. Dyn..

[5] Chen X.Y., Li J.R., Li X.C., Jiang L. (1998). A new step to the mechanism of the enhancement effect of gold nanoparticles on glucose oxidase. Biochem. Biophys. Res. Commun..

[6] Loubeyre P., Zhao S., Canet E., Abidi H., Benderbous S., Revel D. (1997). Ultrasmall superparamagnetic iron oxide particles (AMI 227) as a blood pool contrast agent for MR angiography: Experimental study in rabbits. J. Magn. Reson. Imaging.

[7] Kumar D., Saini N., Jain N., Sareen R., Pandit V. (2013). Gold nanoparticles: An era in bionanotechnology. Expert Opin. Drug Deliv..

[8] Kumar S., Rao R., Kumar A., Mahant S., Nanda S. (2016). Novel Carriers for Coenzyme Q10 Delivery. Curr. Drug Deliv..

[9] Dong Q., Ding Y., Wen B., Wang F., Dong H., Zhang S., Wang T., Yang M. (2012). Improvement of thermal stability of polypropylene using DOPO-immobilized silica nanoparticles. Colloid Polym. Sci..

[10] Sarkar D., Swain S.K., Adhikari S., Reddy B.S., Maiti H.S. (2013). Synthesis, mechanical properties and bioactivity of nanostructured zirconia. Mater. Sci. Eng. C.

[11] Qu M., Mallidi S., Mehrmohammadi M., Truby R., Homan K., Joshi P., Chen Y.-S., Sokolov K., Emelianov S. (2011). Magneto-photo-acoustic imaging. Biomed. Opt. Express.

[12] Chong H., Nie C., Zhu C., Yang Q., Liu L., Lv F., Wang S. (2011). Conjugated Polymer Nanoparticles for Light-Activated Anticancer and Antibacterial Activity with Imaging Capability. Langmuir.

[13] Adwin Jose P., Sankarganesh M., Dhaveethu Raja J., Sukkur Saleem S. (2020). Pyrimidine Derivative Schiff Base Ligand Stabilized Copper and Nickel Nanoparticles by Two Step Phase Transfer Method; in Vitro Anticancer, Antioxidant, Anti-Microbial and DNA Interactions. J. Fluoresc..

[14] Al-Qubaisi M.S., Rasedee A., Flaifel M.H., Ahmad S.H., Hussein-Al-Ali S., Hussein M.Z., Eid E.E., Zainal Z., Saeed M., Ilowefah M. (2013). Cytotoxicity of nickel zinc ferrite nanoparticles on cancer cells of epithelial origin. Int. J. Nanomed..

[15] Qasim M., Asghar K., Dharmapuri G., Das D. (2017). Investigation of novel superparamagnetic Ni0.5Zn0.5Fe2O4@albumen nanoparticles for controlled delivery of anticancer drug. Nanotechnology.

[16] Delong R.K., Comer J., Mathew E.N., Jaberi-Douraki M. (2019). Comparative Molecular Immunological Activity of Physiological Metal Oxide Nanoparticle and its Anticancer Peptide and RNA Complexes. Nanomaterials.

[17] El-Sayed E.-S.R., Abdelhakim H.K., Zakaria Z. (2020). Extracellular biosynthesis of cobalt ferrite nanoparticles by Monascus purpureus and their antioxidant, anticancer and antimicrobial activities: Yield enhancement by gamma irradiation. Mater. Sci. Eng. C.

[18] Jarestan M., Khalatbari K., Pouraei A., Shandiz S.A.S., Beigi S., Hedayati M., Majlesi A., Akbari F., Salehzadeh A. (2020). Preparation, characterization, and anticancer efficacy of novel cobalt oxide nanoparticles conjugated with thiosemicarbazide. 3 Biotech.

[19] Dey C., Ghosh A., Ahir M., Ghosh A., Goswami M.M. (2018). Improvement of Anticancer Drug Release by Cobalt Ferrite Magnetic Nanoparticles through Combined pH and Temperature Responsive Technique. ChemPhysChem.

[20] Park B.J., Choi K.-H., Nam K.C., Ali A., Min J.E., Son H., Uhm H.S., Kim H.-J., Jung J.-S., Choi E.H. (2015). Photodynamic Anticancer Activities of Multifunctional Cobalt Ferrite Nanoparticles in Various Cancer Cells. J. Biomed. Nanotechnol..

[21] Kandil E.I., El-Sonbaty S.M., Moawed F.S., Khedr O.M. (2018). Anticancer redox activity of gallium nanoparticles accompanied with low dose of gamma radiation in female mice. Tumor Biol..

[22] Körhegyi Z., Rózsa D., Hajdu I., Bodnár M., Kertész I., Kerekes K., Kun S., Kollár J., Varga J., Garai I. (2019). Synthesis of 68Ga-Labeled Biopolymer-based Nanoparticle Imaging Agents for Positron-emission Tomography. Anticancer. Res..

[23] Gu C., Li C., Zhang J., Li X., Wang L., Ju Y., Liu Y., Xu Y. (2020). Ultra-effective near-infrared Photothermal therapy for the prostate cancer Nursing care through novel intended and surface tailored photo-responsive Ga-Au@MPS nanovesicles. J. Photochem. Photobiol. B Biol..

[24] Douziech-Eyrolles L., Marchais H., Hervé K., Munnier E., Soucé M., Linassier C., Dubois P., Chourpa I. (2007). Nanovectors for anticancer agents based on superparamagnetic iron oxide nanoparticles. Int. J. Nanomed..

[25] Jahanbani J., Ghotbi M., Shahsavari F., Seydi E., Rahimi S., Pourahmad J. (2020). Selective anticancer activity of superparamagnetic iron oxide nanoparticles (SPIONs) against oral tongue cancer using in vitro methods: The key role of oxidative stress on cancerous mitochondria. J. Biochem. Mol. Toxicol..

[26] Shetake N.G., Kumar A., Pandey B.N. (2019). Iron-oxide nanoparticles target intracellular HSP90 to induce tumor radio-sensitization. Biochim. Biophys. Acta Gen. Subj..

[27] Akhtar S., Rehman S., Almessiere M.A., Alam Khan F., Slimani Y., Baykal A. (2019). Synthesis of Mn_0.5_Zn_0.5_Sm_x_Eu_x_Fe_1.8−2x_O_4_ Nanoparticles via the Hydrothermal Approach Induced Anti-Cancer and Anti-Bacterial Activities. Nanomaterials.

[28] Rehman S., Asiri S.M., Khan F.A., Jermy B.R., Khan H., Akhtar S., Jindan R.A., Khan K.M., Qurashi A. (2019). Biocompatible Tin Oxide Nanoparticles: Synthesis, Antibacterial, Anticandidal and Cytotoxic Activities. Chemistry.

[29] AlAhmari F., Rehman S., Almessiere M., A Khan F., Slimani Y., Baykal A. (2020). Synthesis of Ni_0.5_Co_0.5−x_Cd_x_Fe_1.78_Nd_0.02_O_4_ (x ≤ 0.25) nanofibers by using electrospinning technique induce anti-cancer and anti-bacterial activities. J. Biomol. Struct. Dyn..

[30] Almessiere M., Slimani Y., Rehman S., Khan F.A., Polat E.G., Sadaqat A., Shirsath S.E., Baykal A. (2020). Synthesis of Dy-Y co-substituted manganese-zinc spinel nanoferrites induced anti-bacterial and anti-cancer activities: Comparison between sonochemical and sol-gel auto-combustion methods. Mater. Sci. Eng. C.

[31] Chang Z. (2011). Template Preparation Transition Metal Oxide Hollow Microsphere by Template Method and Study on Its Properties. Master’s Thesis.

[32] Jing W. (2011). Synthesis Several Putamen and Hollow Structure Function Material by Carbon Containing Polysaccharide Microsphere Template Method. Master’s Thesis.

[33] Gu J., Wu G., Zhao X. (2007). Preparation and performance study of high damping cenosphere/epoxy compo site material. Funct. Mater..

[34] Mu G., Pan X., Shen H., Gu M. (2007). Preparation and magnetic properties of composite powders of hollow microspheres coated with barium ferrite. Mater. Sci. Eng. A.

[35] Wang C., Wang J., Li M., Qu X., Zhang K., Rong Z., Xiao R., Wang S. (2016). A rapid SERS method for label-free bacteria detection using polyethylenimine-modified Au-coated magnetic microspheres and Au@Ag nanoparticles. Analyst.

[36] Huang Y.-F., Wang Y.-F., Yan X.-P. (2010). Amine-functionalized magnetic nanoparticles for rapid capture and removal of bacterial pathogens. Environ. Sci. Technol..

[37] Li Z., Ma J., Ruan J., Zhuang X. (2019). Using positively charged magnetic nanoparticles to capture bacteria at ultralow concentration. Nanoscale Res. Lett..

[38] Rethinasabapathy M., Vilian A.E., Hwang S.K., Kang S.-M., Cho Y., Han Y.-K., Rhee J.-K., Huh Y.S. (2021). Cobalt ferrite microspheres as a biocompatible anode for higher power generation in microbial fuel cells. J. Power Sources.

[39] Chen P., Cui B., Bu Y., Yang Z., Wang Y. (2017). Synthesis and characterization of mesoporous and hollow-mesoporous M_x_Fe_3−x_O_4_ (M = Mg, Mn, Fe, Co, Ni, Cu, Zn) microspheres for microwave-triggered controllable drug delivery. J. Nanopart. Res..

[40] Alam Khan F., Akhtar S., Almohazey D., AlOmari M., Almofty S., Eliassari A. (2018). Fluorescent magnetic submicronic polymer (FMSP) nanoparticles induce cell death in human colorectal carcinoma cells. Artif. Cells Nanomed. Biotechnol..

[41] Alam Khan F., Akhtar S., Almohazey D., AlOmari M., Almofty S. (2018). Extracts of Clove (Syzygium aromaticum) Potentiate FMSP-Nanoparticles Induced Cell Death in MCF-7 Cells. Int. J. Biomater..

[42] Baig U., Ansari M.A., Gondal M.A., Akhtar S., Khan F.A., Falath W.S. (2020). Single step production of high-purity copper oxide-titanium dioxide nanocomposites and their effective antibacterial and anti-biofilm activity against drug-resistant bacteria. Mater. Sci. Eng. C.

[43] Rehman S., Almessiere M., Khan F., Korkmaz A.D., Tashkandi N., Slimani Y., Baykal A. (2020). Synthesis and biological characterization of Mn_0.5_Zn_0.5_Eu_x_Dy_x_Fe_1.8-2x_O_4_ nanoparticles by sonochemical approach. Mater. Sci. Eng. C.

[44] Rehman S., Almessiere M.A., Tashkandi N., Baykal A., Slimani Y., Jermy R., Ravinayagam V., Yaman C. (2019). Fabrication of Spinel Cobalt Ferrite (CoFe_2_O_4_) Nanoparticles with Unique Earth Element Cerium and Neodymium for Anticandidal Activities. Chemistry.

[45] El Rayes S.M., Aboelmagd A., Gomaa M., Ali I.A.I., Fathalla W., Pottoo F.H., Khan F.A. (2019). Convenient Synthesis and Anticancer Activity of Methyl 2-[3-(3-Phenyl-quinoxalin-2-ylsulfanyl)propanamido]alkanoates and N-Alkyl 3-((3-Phenyl-quinoxalin-2-yl)sulfanyl)propanamides. ACS Omega.

[46] Aldakheel R.K., Rehman S., Almessiere M.A., Khan F.A., Gondal M.A., Mostafa A., Baykal A. (2020). Bactericidal and In Vitro Cytotoxicity of Moringa oleifera Seed Extract and Its Elemental Analysis Using Laser-Induced Breakdown Spectroscopy. Pharmaceuticals.

[47] Khan F.A., Lammari N., Muhammad Siar A.S., Alkhater K.M., Asiri S., Akhtar S., Almansour I., Alamoudi W., Haroun W., Louaer W. (2020). Quantum dots encapsulated with curcumin inhibit the growth of colon cancer, breast cancer and bacterial cells. Nanomedicine.

[48] Veeralakshmi S., Nehru S., Sabapathi G., Arunachalam S., Venuvanalingam P., Kumar P., Anusha C., Ravikumar V. (2015). Single and double chain surfactant–cobalt (III) complexes: The impact of hydrophobicity on the interaction with calf thymus DNA, and their biological activities. RSC Adv..

[49] Nguyen K.T., Shukla K.P., Moctezuma M., Braden A.R.C., Zhou J., Hu Z., Tang L. (2008). Studies of the cellular uptake of hydrogel nanospheres and microspheres by phagocytes, vascular endothelial cells, and smooth muscle cells. J. Biomed. Mater. Res. Part A.

[50] Do D.P., D’Souza M.J., Enriquez G.G., Rizvi S.A. (2013). Formulation and evaluation of drug-loaded targeted magnetic microspheres for cancer therapy. Int. J. Nanomed..

[51] Patra J.K., Das G., Fraceto L.F., Campos E.V.R., Rodriguez-Torres M.D.P., Acosta-Torres L.S., Diaz-Torres L.A., Grillo R., Swamy M.K., Sharma S. (2018). Nano based drug delivery systems: Recent developments and future prospects. J. Nanobiotechnol..

[52] Chohan Z.H., Khan K.M., Supuran C.T. (2004). Synthesis of antibacterial and antifungal cobalt (II), copper (II), nickel (II) and zinc (II) complexes with bis-(1,1′-disubstituted ferrocenyl) thiocarbohydrazone and bis-(1,1′-disubstituted ferrocenyl) carbohydrazone. Appl. Organomet. Chem..

[53] Shah A.H., Rather M.A. (2021). Effect of Thermal Treatment on the Phase Composition and Surface Properties of WO_3_-TiO_2_ Nanocomposites Synthesized via Hydro-Thermal Method. Chemistry.

[54] Nagy M., Szemán-Nagy G., Kiss A., Nagy Z.L., Tálas L., Rácz D., Majoros L., Tóth Z., Szigeti Z.M., Pócsi I. (2020). Antifungal activity of an original amino-isocyanonaphthalene (ICAN) compound family: Promising broad spectrum antifungals. Molecules.

